# Outbreak of Pathogenic *Streptococcus equi* subsp. *zooepidemicus* in Guinea Pigs Farms of The Andean Region

**DOI:** 10.3390/pathogens12030445

**Published:** 2023-03-12

**Authors:** Luis M. Jara, Jose Angulo-Tisoc, Luis G. Giménez-Lirola, Ganwu Li, Roy Andrade, Javier Mamani

**Affiliations:** 1 Facultad de Medicina Veterinaria y Zootecnia, Universidad Peruana Cayetano Heredia, Lima 15102, Peru; 2 Facultad de Medicina Veterinaria, Universidad Nacional Mayor de San Marcos, Lima 15021, Peru; 3 College of Veterinary Medicine, Iowa State University, Ames, IA 50011, USA

**Keywords:** *Streptococcus*, guinea pigs, zoonoses, outbreak, lymphadenitis

## Abstract

*Streptococcus zooepidemicus* is an emerging zoonotic pathogen involved in septicemic infections in humans and livestock. Raising guinea pigs in South America is an important economic activity compared to raising them as pets in other countries. An outbreak of severe lymphadenitis was reported in guinea pigs from farms in the Andean region. *S. zooepidemicus* was isolated from multiple cervical and mandibular abscesses. Isolate was characterized by multilocus sequence typing and phylogenetic analysis. This is the first molecular characterization of a highly pathogenic strain, showing major important virulence factors such as the M-like protein genes *szP* and *mlpZ*, the fimbrial subunit protein gene *fszF*, and the protective antigen-like protein gene *spaZ.* Additionally, this guinea pig strain was phylogenetically related to equines but distant from zoonotic and pig isolates reported in other countries.

## 1. Introduction

*Streptococcus equi* subsp. *zooepidemicus* (*S. zooepidemicus*) is a zoonotic, Gram-positive bacterium that causes mainly septicemic infections in pets, livestock, and humans worldwide. It is frequently isolated as a commensal pathogen of horses, the natural reservoir, and causes purulent respiratory (severe pneumonia) and uterine infections. It has also been reported in dogs and South American camelids [1,2]. *S. zooepidemicus* opportunistically produces disease in situations of viral infection, stress, or tissue injury. Its widely ranging pathogenicity includes mastitis in cattle and goats; pneumonia, septicemia, and wound infections in lambs, puppies, and greyhounds; septicemia in chickens, dolphins, swine, guinea pigs, chinchillas, and monkeys [3,4]. In humans, it is associated with nephritis outbreaks and other infections (meningitis, endocarditis) often traced back to the consumption of contaminated dairy products [5]. Zoonoses are also caused by contact with sick animals [4].

In the Andean region, family and commercial raising of guinea pigs for meat consumption is an important economic activity. Farms may have poor sanitary conditions and low biosecurity, which increase the risk of infectious diseases and subsequent economic losses. Streptococcal lymphadenitis in guinea pigs is an infection of the cervical lymph nodes caused by *S. zooepidemicus*, which invades the abraded oral mucosa [5]. Oral abrasions caused by overgrown teeth, abrasive feed, or bite wounds lead to invasion of the bacteria into deeper tissues and cervical lymph nodes, which become abscessed [6].

β-hemolytic streptococcal isolations have been reported from affected and dead guinea pigs of experimental and commercial farms [7,8] as well as in pets [9]. Preventive measures include autogenous bacterins, which are administered in some farms with unknown results of immunogenicity. However, there are no previous reports characterizing *S. zooepidemicus* virulent genes and phylogenetic analysis at the species level with circulant isolates from other countries and hosts.

This report describes the identification and molecular characterization of virulent *S. zooepidemicus* causing a severe lymphadenitis outbreak in a guinea pig farm in the Andean region.

## 2. Case

**Figure 1:**
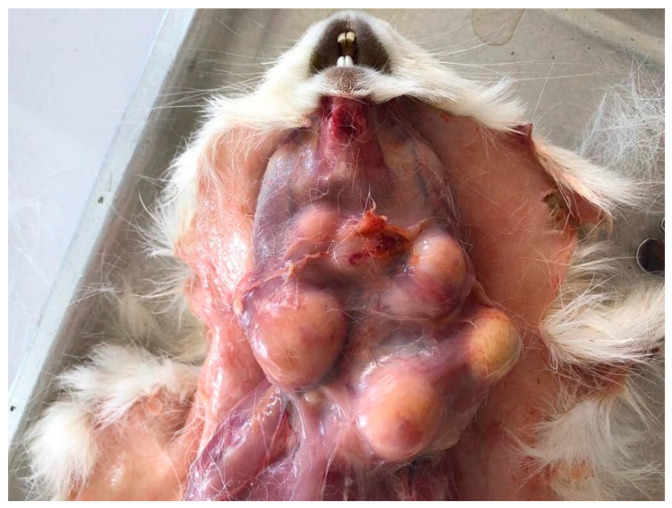
Macroscopic lesions of lymphadenitis in guinea pigs. Multiple purulent abscesses of the cervical, mandibular, and retropharyngeal lymph nodes in affected guinea pig.

At the end of 2019, a severe outbreak of presumptive infectious lymphadenitis was reported in a small farm of guinea pigs (Andina and Peru breeds) in the south of the Peruvian Highlands, at 3554 m above sea level. Animals were reared in wooden cages using the natural ground as a contact surface, distributed in pens in a proportion of 5:1 of females and males, with ages of 6–40 months and an average weight of 0.8 kg. The feed was based on forages such as alfalfa and ryegrass, and the concentrate was based on oats, corn flour, and barley. Clinical signs in affected animals included cervical and mandibular swelling. Lymphadenitis was observed in 70% of animals (175/250), yet only 23% (40/175) showed severe illness such as cervical or mandibular swelling (more than one abscess of 5 cm at physical examination) and lethargy. Due to the high morbidity, farmers euthanized and discarded the most severely affected animals; however, 10 of these animals were necropsied for further diagnostic investigation. Gross examination showed multiple cervical and mandibular abscesses in several animals (Figure 1). Affected guinea pigs with mild clinical signs were also euthanized and discarded because of transmission risk, the difficulty of treating abscesses, and the potential risk of zoonotic transmission.

## 3. Materials and Methods

Five swabs from a pool of purulent exudate from guinea pigs with severe illness (10 animals) were taken for bacterial isolation using Sheep Blood agar and the assessment of antimicrobial susceptibility in vitro using a disk diffusion method in Mueller–Hinton agar [10]. Identification of pure colonies was performed by macroscopic growth observation, Gram staining, catalase, and the salt tolerance (6.5% NaCl) test. Suspected β-hemolytic streptococci were selected for preliminary PCR identification and MLST analysis for *S. zooepidemicus*.

Genomic DNA was extracted and purified from an overnight single colony, isolated, and cultured in Brain Heart Infusion at 37 °C using a commercial GeneJET Genomic DNA Purification Kit (Thermo Fisher Scientific, Waltham, MA, USA), following the manufacturer’s guidelines. DNA quality was determined by NanoDrop (Thermo Fisher Scientific, Waltham, MA, USA), and accurate concentration was measured by a Qubit fluorometer using a double-stranded DNA high-sensitivity (dsDNA HS) kit (Thermo Fisher Scientific, Waltham, MA, USA). *S. zooepidemicus* was determined by end-point PCR amplification of the partial *szP* gene [11]. Indexed genomic libraries were prepared from one pure bacterium colony of one isolate by using the Nextera XT DNA library prep kit (Life Technologies) for subsequent sequencing at the Illumina MiSeq platform (Illumina, San Diego, CA, USA) with a 250 × 2 read length.

Genome assembly and phylogenetic analysis were performed as previously described [12]. The genome sequencing and assembly data were deposited at NCBI under BioProject Accession: PRJNA791851. Single nucleotide polymorphisms (SNPs) of this and other 48 *S. zooepidemicus* isolates from the previous study were identified by running kSNP3 in standard mode. The optimal k-mers size was calculated by the Kchooser program kSNP (CA, USA, v. 3.0), and the whole-genome phylogeny was analyzed based on identified core genome SNPs [13]. Sequence type (ST) based on 7 highly conserved housekeeping genes (*arc*, *nrdE*, *proS*, *spi*, *tdk*, tpi, and *yqiL*) [14] was assigned for each *S. zooepidemicus* genome according to the PubMLST *S. zooepidemicus* database at http://pubmlst.org/szooepidemicus (accessed on 14 November 2021) [15]. Putative virulence genes were retrieved from genome sequences according to previous publications [16,17,18,19,20]. Tree Of Life iTOL at https://itol.embl.de (accessed on 14 November 2021) [21] was used for display, manipulation, and annotation on the base of the core SNPs tree.

Tissues surrounded by abscesses were also fixed in 10% formalin for histopathological examination.

## 4. Results

Bacterial culture from all the swabs collected resulted in the isolation of pure pinpoint and circular small colonies with complete β-hemolysis (five isolates). Gram staining showed Gram-positive cocci arranged in pairs or chains with the capsule. In vitro, antibiotic sensitivity testing indicated sensitivity to tetracycline, enrofloxacin, penicillin, ceftiofur, sulfamethoxazole-trimethoprim, and amoxicillin-clavulanate, common antibiotics used in small mammals.

**Figure 2:**
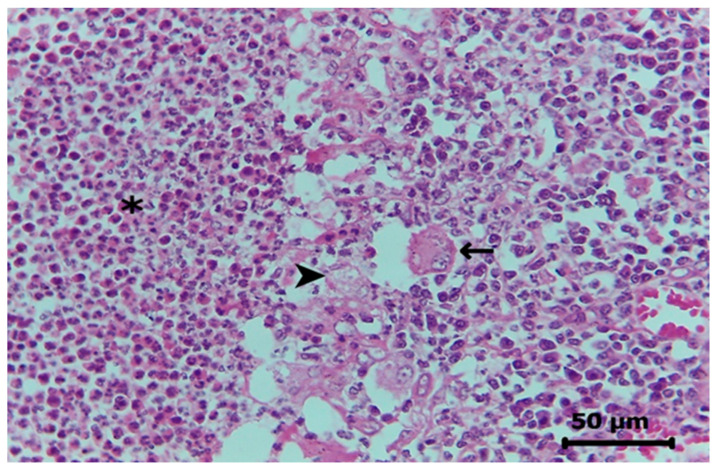
Histopathologically, normal lymph node architecture is lost and replaced by a massive amount of degenerated heterophiles mixed with cellular debris (**asterisk**) and the presence of many foamy macrophages (**arrowhead**) and multinucleated giant cell macrophages (**arrow**). Hematoxylin and eosin staining, original magnification x 40.

Histopathologically, a loss of lymphoid architecture was replaced by heterophiles or macrophages and granulation tissue (Figure 2), which were associated with intralesional Gram-positive cocci. The diagnosis was chronic, severe pyogranulomatous lymphadenitis.

**Figure 3:**
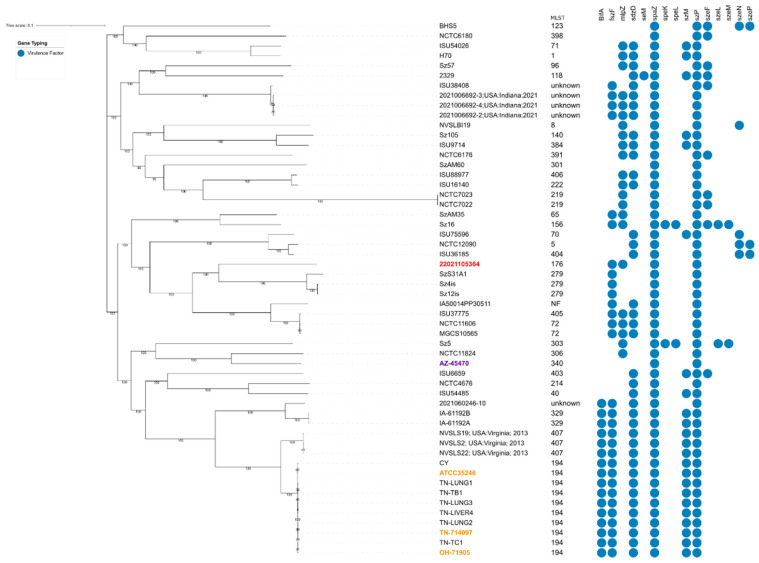
Genetic characterization of pathogenic *S. zooepidemicus* isolate from guinea pigs affected with lymphadenitis. Whole-genome sequence-based phylogenetic analysis was conducted using SNPs located in all tested *S. zooepidemicus* genomes to generate a core SNP parsimony tree. The branches of the tree are proportional to the distance between the isolates. *S. zooepidemicus* isolate 22021105364 from guinea pigs in Peru is highlighted in red; outbreak isolates of *S. zooepidemicus* from Ohio (OH-71905) and Tennessee (TN-714097) and the ATCC35246 are highlighted in orange; and the outbreak-unrelated isolate AZ-45470 of *S. zooepidemicus* from Arizona is highlighted in violet.

Further molecular diagnostics of the isolates confirmed the identification of *S. zooepidemicus* in all swab cultures. The guinea pig isolate (MLST type 176) belongs nearly to MLST type 279, closely related to the equine isolates SzS31A1, Sz4is, and Sz12is, but genetically distant from three isolates from human cases with guinea pig exposure (NVSLS19, NVSLS2, and NVSLS22) [9]. In addition, this isolate is genetically distant from the highly virulent Ohio, Tennessee, and Indiana isolates associated with high mortality events in pigs in the United States [12], yet distant from the ATCC 35246 strain associated with a pig outbreak reported in China [22]. The presence of 15 putative virulence genes previously reported in *S. zooepidemicus* [16,17,18,19,20] was also investigated in the current study. Similar to the Ohio, Tennessee, and Indiana isolates (references) and the ATCC 35246 strain, the *S. zooepidemicus* isolated from guinea pigs during the outbreak described herein possessed the M-like protein genes *szP* and *mlpZ*, the fimbrial subunit protein coding gene *fszF*, and the protective antigen-like protein coding gene *spaZ* (Figure 3). However, it was negative for the second M-like protein gene, *szM* or the newly identified Fic domain-containing protein gene, *bifA*. The superantigens *szeF*, *szeL*, *szeM*, *szeN*, *szeP,* and some antimicrobial resistance genes were all absent.

## 5. Discussion

*S. zooepidemicus* is an opportunistic commensal frequently isolated from the respiratory tract mucosa in horses. However, molecular diagnostic and genetic analyses of strains from different species and regions have not been well characterized. This bacterium is one of the main infectious agents circulating in family- and commercially- raised guinea pigs in the Andean region. Previous reports showed an isolation frequency of 20% in guinea pigs, often associated with characteristic pathologic lesions (i.e., cervical purulent lymphadenitis) from the central highlands of Peru, and between 70% and 100% in other regions of Peru. However, other opportunistic agents, including *Staphylococcus*, *Klebsiella*, *Corynebacterium*, and non-hemolytic *Streptococcus*, can also be isolated, especially in adult guinea pigs [8].

Zoonotic cases of *S. zooepidemicus* in humans have been described in different clinical reports, including meningitis, arthritis, glomerulonephritis, and pneumonia [23]. Most cases were due to the consumption of unpasteurized milk, direct contact with equine nasal secretions, or pets living close to horses [24]. Some cases in the United States were associated with guinea pigs kept as domestic pets [9]. By contrast, some countries in South America raise these animals for local consumption and export their meat to international markets. For instance, Peru has a higher population of guinea pigs, with more than 800 thousand farmers and 17 million animals [25]. Moreover, mixed breeding of different livestock animals is also common in this country, enabling *S. zooepidemicus* to adapt naturally to different hosts according to the specific geographical area. For instance, some swine *S. zooepidemicus* strains from China, Canada, and the United States associated with high mortality form a genetic cluster (ST194) together with three human isolates from guinea pigs’ exposition in the United States [12,26]. In the present report, the isolated Peruvian *S. zooepidemicus* strain was closely related to equine isolates but distant from the above-mentioned zoonotic isolates of high mortality identified in pigs and guinea pigs. Besides, only one official communication of streptococcal zoonoses caused by consumption of unpasteurized milk from goats has been reported in Peru [27], which did not include a comprehensive molecular characterization. Despite the limited information available, the evidence of the circulation of a virulent *S. zooepidemicus* in guinea pig farms should be a public health concern because some strains, such as ST194, have been reported in several cases of human septicemia with up to 33% mortality in Thailand from 2005 to 2020 [28].

The putative virulent genes M-like protein *szP* and *mlpZ*, fimbrial subunit protein coding *fszF*, and protective antigen-like protein coding *spaZ* identified in the present study were also reported in an *S. zooepidemicus* strain that caused zoonotic necrotizing myositis in a farmer from Norway [18] and in previous outbreaks with high mortality (up to 50%) in swine farms in the United States [12]. As suggested by this previous study, it would be interesting to determine whether one or more specific genes contribute to the virulence of *S. zooepidemicus* in different hosts, including humans and guinea pigs from different areas. For instance, ST194, ST-236, and ST-72 strains are adapted to pigs, ruminants, and humans, respectively; meanwhile, ST-1, ST-2, or ST-209 strains have been commonly associated with respiratory infections in horses. Cumulative evidence provides an opportunity to identify potential genes influencing the host and the tissue specificity required for *S. zooepidemicus* to cause disease [29]. In addition, genomic determinants of antimicrobial resistance were not fully evaluated in our strain, even though beta-hemolytic *Streptococcus* sp. from lymphadenitis in guinea pigs showed a low in vitro antimicrobial resistant pattern for common antibiotics (enrofloxacin and trimethoprim/sulfamethoxazole) used by farmers from the outbreak area of this study [8].

Although streptococcal infections have been described in guinea pigs as laboratory animal models or pets since 1907, this report is the first molecular identification and characterization of pathogenic *S. zooepidemicus* associated with high morbidity outbreaks from naturally infected guinea pigs from farms for meat purposes in the Andean region. Preventive measures for streptococcal lymphadenitis include floor disinfection of farms, quarantine of newly infected or suspected animals, avoiding overcrowding of animals in cages, and most recently, the use of vaccines. In horses, additional risk factors such as coinfection with the influenza virus and thermal and transportation stress have been associated with severe cases of respiratory disease by *S. zooepidemicus* [4]. Due to high morbidity and the difficulty of draining abscesses under field conditions in highly populated guinea pig farms, farmers identify and discard clinically affected animals (partial/targeted depopulation or precision removal) as a control measure. Overall, biosecurity measures in guinea pig farms and slaughterhouses are key to avoiding the potential risk of zoonotic transmission from animals with purulent lymphadenitis.

Molecular characterization is relevant because it allows the screening of native virulent strains circulating in farms for comparative model studies, the identification of virulent strains, susceptibility across species, zoonotic potential, and the development and assessment of effective vaccines, among others. For instance, it has been reported that the *S. zooepidemicus* guinea pig strain causes a similar severe systemic disease in pigs; however, the horse strain appeared to be less virulent for pigs [30]. Moreover, pig vaccination with an *S. zooepidemicus* bacterium did not provide protection from disease and did not generate high titters of antibodies [31]. This is noteworthy because a commercial autogenous bacterin has been used in some farms in Peru, but specific information about its potential immunity against virulent *S. zooepidemicus* in guinea pigs remains unclear.

This report highlights the importance of genomic surveillance in guinea pig farms for screening of virulence factors towards the investigation of the molecular epidemiology of *S. zooepidemicus* pathotypes from different hosts and possible routes of transmission during outbreaks or zoonotic events, which may also be useful for the development of specific and protective vaccines.

## 6. Conclusions

We report the first molecular characterization of a high-pathogenic *S. zooepidemicus* isolate from guinea pig farms in South America. This isolate showed major virulent genes and was closely related to horses’ isolates but distant from pigs and human cases, with guinea pig exposure worldwide.

## Data Availability

The genome sequencing and assembly data were deposited under BioProject Accession: PRJNA791851 is available from NCBI’s Sequence Read Archive at https://www.ncbi.nlm.nih.gov/bioproject/PRJNA791851 (accessed on 23 November 2021).

## References

[1] Corpa J.M., Carvallo F., Anderson M.L., Nyaoke A.C., Moore J.D., Uzal F.A. (2018). *Streptococcus equi* subspecies *zooepidemicus* Septicemia in Alpacas: Three Cases and Review of the Literature. J. Vet. Diagn. Invest..

[2] Priestnall S., Erles K. (2011). *Streptococcus zooepidemicus*: An Emerging Canine Pathogen. Vet. J..

[3] Mitchell C.M., Johnson L.K., Crim M.J., E Wiedmeyer C., Pugazhenthi U., Tousey S., Tollin D.J., Habenicht L.M., Fink M.K., Fong D.L. (2020). Diagnosis, Surveillance and Management of *Streptococcus equi* subspecies *zooepidemicus* Infections in Chinchillas (*Chinchilla lanigera*). Comp. Med..

[4] Prescott J.F., MacInnes J.I., Van Immerseel F., Boyce J.D., Rycroft A.N., Vázquez-Boland J.A. (2023). Pathogenesis of Bacterial Infections in Animals.

[5] Mayer J., Donnelly T.M. (2013). Clinical veterinary advisor: Birds and exotic pets.

[6] Quesenberry K.E., Orcutt C.J., Mans C., Carpenter J.W. (2021). Ferrets, Rabbits, and Rodents: Clinical Medicine and Surgery.

[7] Killerby M., Huamán M., Chauca L. (2020). Identificación de Los Agentes Bacterianos Relacionados con Mortalidad en Cuyes Reproductores de Crianza Intensiva. Salud Tecnol. Vet..

[8] Angulo-Tisoc J.M., Jara L.M., Pacheco J.I., Pezo D. (2021). Frecuencia de Agentes Bacterianos Asociados a Mortalidad en Cuyes de Centros de Crianza Familiar-Comercial en Canchis, Cusco. Rev. de Investig. Vet. del Peru.

[9] Gruszynski K., Young A., Levine S.J., Garvin J.P., Brown S., Turner L., Fritzinger A., Gertz R.E., Murphy J.M., Vogt M. (2015). *Streptococcusequi* subsp. *Zooepidemicus* Infections Associated with Guinea Pigs. Emerg. Infect. Dis..

[10] CLSI (2020). VET01S Performance Standards for Antimicrobial Disk and Dilution Susceptibility Tests for Bacteria Isolated from Animals.

[11] A Walker J., Timoney J.F. (1998). Molecular Basis of Variation in Protective SzP Proteins of *Streptococcus zooepidemicus*. Am. J. Vet. Res..

[12] Chen X., Resende-De-Macedo N., Sitthicharoenchai P., Sahin O., Burrough E., Clavijo M., Derscheid R., Schwartz K., Lantz K., Robbe-Austerman S. (2020). Genetic Characterization of *Streptococcus equi* subspecies *zooepidemicus* associated with High Swine Mortality in the United States. Transbound. Emerg. Dis..

[13] Gardner S.N., Slezak T., Hall B.G. (2015). kSNP3.0: SNP Detection and Phylogenetic Analysis of Genomes Without Genome Alignment or Reference Genome. Bioinformatics.

[14] Webb K., Jolley K.A., Mitchell Z., Robinson C., Newton J.R., Maiden M.C.J., Waller A. (2008). Development of an Unambiguous and Discriminatory Multilocus Sequence Typing Scheme for the *Streptococcus zooepidemicus* Group. Microbiology.

[15] A Jolley K., Chan M.-S., Maiden M.C.J. (2004). mlstdbNet—Distributed Multi-Locus Sequence Typing (MLST) Databases. BMC Bioinform..

[16] Alber J., El-Sayed A., Estoepangestie S., Lämmler C., Zschöck M. (2005). Dissemination of the Superantigen Encoding Genes *seeL, seeM, szeL* and *szeM* in *Streptococcus equi* subsp. *equi* and *Streptococcus equi* subsp. *zooepidemicus*. Vet. Microbiol..

[17] Bergmann R., Jentsch M.-C., Uhlig A., Müller U., van der Linden M., Rasmussen M., Waller A., von Köckritz-Blickwede M., Baums C.G. (2019). Prominent Binding of Human and Equine Fibrinogen to *Streptococcus equi* subsp. *zooepidemicus* is Mediated by Specific SzM Types and Is a Distinct Phenotype of Zoonotic Isolates. Infect. Immun..

[18] Kittang B.R., Pettersen V.K., Oppegaard O., Skutlaberg D.H., Dale H., Wiker H.G., Skrede S. (2017). Zoonotic Necrotizing Myositis Caused by *Streptococcus equi* subsp. *zooepidemicus* in a Farmer. BMC Infect. Dis..

[19] Ma Z., Peng J., Yu D., Park J.S., Lin H., Xu B., Lu C., Fan H., Waldor M.K. (2019). A streptococcal Fic Domain-Containing Protein Disrupts Blood-Brain Barrier Integrity by Activating Moesin in Endothelial Cells. PLoS Pathog..

[20] Rash N., Robinson C., DeSouza N., Nair S., Hodgson H., Steward K., Waller A., Paillot R. (2014). Prevalence and Disease Associations of Superantigens *szeF, szeN* and *szeP* in the *S. zooepidemicus* Population and Possible Functional Redundancy of *szeF*. Res. Vet. Sci..

[21] Letunic I., Bork P. (2019). Interactive Tree Of Life (iTOL) v4: Recent Updates and New Developments. Nucleic Acids Res..

[22] Ma Z., Geng J., Zhang H., Yu H., Yi L., Lei M., Lu C.-P., Fan H.-J., Hu S. (2011). Complete Genome Sequence of *Streptococcus equi* subsp. *zooepidemicus* Strain ATCC 35246. J. Bacteriol..

[23] Abbott Y., Acke E., Khan S., Muldoon E.G., Markey B.K., Pinilla M., Leonard F.C., Steward K., Waller A. (2010). Zoonotic Transmission of I subsp. *Zooepidemicus* from a Dog to a Handler. J. Med. Microbiol..

[24] Rajasekhar A., Clancy C.J. (2010). Meningitis Due to Group C *Streptococcus*: A Case Report and Review of the Literature. Scand. J. Infect. Dis..

[25] (2020). Instituto Nacional de Innovacion Agraria. https://www.inia.gob.pe/2020-nota-105/.

[26] Costa M.D.O., Lage B. (2020). *Streptococcus equi* subspecies *zooepidemicus* and Sudden Deaths in Swine, Canada. Emerg. Infect. Dis..

[27] Mori N., Guevara J.M., Tilley D.H., Briceno J.A., Zunt J.R., Montano S.M. (2013). *Streptococcus equi* subsp. *zooepidemicus* Meningitis in Peru. J. Med. Microbiol..

[28] Kerdsin A., Chopjitt P., Hatrongjit R., Boueroy P., Gottschalk M. (2021). Zoonotic Infection and Clonal Dissemination of *Streptococcus equi* subspecies *zooepidemicus* Sequence Type 194 Isolated from Humans in Thailand. Transbound. Emerg. Dis..

[29] Waller A.S., Wilson H. Streptococcus zooepidemicus: Commensal or Pathogen?. Proceedings of the 67th Annual Convention of the American Association of Equine Practitioners.

[30] Hau S.J., Lantz K., Stuart K.L., Sitthicharoenchai P., Macedo N., Derscheid R.J., Burrough E.R., Robbe-Austerman S., Brockmeier S.L. (2022). Replication of *Streptococcus equi* subspecies *zooepidemicus* Infection in Swine. Vet. Microbiol..

[31] Hau S.J., Buckley A., Brockmeier S.L. (2022). Bacterin Vaccination Provides Insufficient Protection Against *Streptococcus equi* subspecies *zooepidemicus* Infection in Pigs. Front. Vet. Sci..

